# An apple rootstock overexpressing a peach CBF gene alters growth and flowering in the scion but does not impact cold hardiness or dormancy

**DOI:** 10.1038/hortres.2016.6

**Published:** 2016-03-09

**Authors:** Timothy S Artlip, Michael E Wisniewski, Rajeev Arora, John L Norelli

**Affiliations:** 1 USDA-ARS, Appalachian Fruit Research Station, Kearneysville, WV, USA; 2 Department of Horticulture, Iowa State University, Ames, IA, USA

## Abstract

The C-repeat binding factor (CBF) transcription factor is involved in responses to low temperature and water deficit in many plant species. Overexpression of *CBF* genes leads to enhanced freezing tolerance and growth inhibition in many species. The overexpression of a peach *CBF* (*PpCBF1*) gene in a transgenic line of own-rooted apple (*Malus*×*domestica*) M.26 rootstock (T166) trees was previously reported to have additional effects on the onset of dormancy and time of spring budbreak. In the current study, the commercial apple cultivar ‘Royal Gala’ (RG) was grafted onto either non-transgenic M.26 rootstocks (RG/M.26) or transgenic M.26 (T166) rootstocks (RG/T166) and field grown for 3 years. No *PpCBF1* transcript was detected in the phloem or cambium of RG scions grafted on T166 rootstocks indicating that no graft transmission of transgene mRNA had occurred. In contrast to own-rooted T166 trees, no impact of *PpCBF1* overexpression in T166 rootstocks was observed on the onset of dormancy, budbreak or non-acclimated leaf-cold hardiness in RG/T166 trees. Growth, however, as measured by stem caliper, current-year shoot extension and overall height, was reduced in RG/T166 trees compared with RG/M.26 trees. Although flowering was evident in both RG/T166 and RG/M.26 trees in the second season, the number of trees in flower, the number of shoots bearing flowers, and the number of flower clusters per shoot was significantly higher in RG/M.26 trees than RG/T166 trees in both the second and third year after planting. Elevated levels of *RGL* (*DELLA*) gene expression were observed in RG/T166 trees and T166 trees, which may play a role in the reduced growth observed in these tree types. A model is presented indicating how *CBF* overexpression in a rootstock might influence juvenility and flower abundance in a grafted scion.

## Introduction

C-repeat binding factor (CBF)/drought response element binding (DREB) proteins function as transcription factors that regulate plant responses to low temperature and water deficit. They represent a sub-family within the larger AP2-domain family of transcription factors. *CBF* genes have been identified in a wide range of herbaceous^1–4^ and woody plants.^5–9^ At least five *CBF* genes^9^ have been identified in both apple (*Malus*×*domestica*) and peach (*Prunus persica*) that exhibit a variety of patterns of expression in response to low temperatures.^10–12^

Overexpression of *CBF* genes has been demonstrated to increase freezing tolerance in several plant systems, including homologous and/or heterologous expression in Arabidopsis and tobacco,^2^ tomato,^13^ potato,^14^ poplar,^6^ grape,^15^ *Eucalyptus*^8^ and apple.^10^ Growth inhibition in both herbaceous^16–19^ and perennial, woody plants has also been observed, including *Eucalyptus*,^8^ grape^15^ and apple.^10,12,20^ Wisniewski *et al.* ^10^ also reported that apple trees (M.26 rootstock) overexpressing the peach (*Prunus persica*) *PpCBF1* gene, in contrast to non-transformed plants, responded to short-day photoperiods by entering dormancy and exhibiting premature leaf senescence. The short-day-response was unexpected as apple has been shown to respond to low temperature rather than photoperiod cues for regulating the onset of dormancy.^21^ In a subsequent report, Artlip *et al.*^20^ noted that self-rooted, field-planted apple trees overexpressing *PpCBF1* exhibited all the characteristics observed in growth chamber studies, including enhanced cold hardiness in acclimated and non-acclimated leaves, growth inhibition, early dormancy and premature leaf senescence. They also observed that spring budbreak was delayed in the transgenic trees and that the overall phenotype of the transgenic trees was stable over at least three seasons of growth.

Wisniewski *et al.*^12^ examined several genes (*RGL, DAM and EBB*) that had been reported to be correlated with growth and dormancy in other woody species and compared their level of expression with non-transformed trees. *RGL* (*DELLA*) genes are involved with gibberellic acid (GA) signaling, acting to suppress GA-mediated growth.^22^ Achard *et al.*^23^ reported that overexpression of CBF proteins resulted in decreased GA levels and increased RGL/DELLA protein levels, thus inhibiting growth. Wisniewski *et al.*^12^ showed that two of six apple *RGL*/*DELLA* genes exhibited temporally elevated expression levels over the course of a year in trees overexpressing *PpCBF1* compared with non-transformed trees. Genes associated with dormancy in perennial, woody plants were also examined.^12^ Although the expression levels of three *DAM* (*Dormancy-associated MADS-box*) genes did not differ between transgenic and non-transgenic trees in bark tissues during most of the season, distinct differences were observed in buds just before and during budbreak. More specifically, two *MdDAM* genes exhibited higher expression levels in transgenic trees. Elevated levels of *EBB1* (*Early Budbreak 1*) gene transcripts have been reported to be highly correlated with budbreak in poplar.^24^ Elevated expression levels of the apple homolog, *MdEBB1*, were also shown to correspond to budbreak in field-grown apple trees. Increases in *MdEBB1* were temporally delayed in the *PpCBF1* overexpressing trees, correlating well with the observed delay in budbreak in those trees.

Rootstocks are widely used in perennial fruit crops to convey a range of horticultural traits, with the main influence being on growth control or dwarfing. As the parent material that was used for the overexpression of *PpCBF1* was an apple rootstock (M.26), a logical extension of the previous studies was to determine what physiological and morphological effects would be transmitted to any scions that were grafted on the transgenic rootstock. Although the impact of rootstocks on scions is in part regulated via hormones,^25,26^ graft transmission of mRNAs from transgenic, herbaceous donor plants to herbaceous-grafted plants has also been reported,^27^ with transmission of silencing RNAs (siRNAs) across the graft union recently reported in cherry.^28^ In addition, transmission of transgenic proteins across the graft union has also been reported in woody plants.^29,30^ In the current study, ‘Royal Gala’ was bud-grafted onto either non-transgenic M.26 or transgenic M.26 (T166) rootstocks (constitutively expressing a peach *CBF*, *PpCBF1*). Self-bud-grafted non-transgenic M.26 and T166 were also created along with own-rooted non-transgenic M.26 and T166 rootstocks. Six-month-old trees were planted and physiological, phenological and gene expression data acquired over 3 years.

## Materials and methods

### Plant material

The peach (*Prunus persica*) *PpCBF1* gene was cloned and an overexpressing transgenic line, T166, was developed as reported in Wisniewski *et al.*^10^ Explants of non-transformed ‘Royal Gala’, non-transformed M.26 and transformed T166 were propagated in tissue culture as described by Norelli *et al.*^31^ and Ko *et al.*,^32^ with root induction as described by Bolar *et al.*^33^ After establishment, propagated trees were grown in an environmental chamber and greenhouse as described in Wisniewski *et al.*^10^ Buds of ‘Royal Gala’ were T-budded onto either M.26 or T166 trees. The grafted plants were grown for at least 6 months before being planted in the field. Own-rooted M.26 and T166 rootstocks were grown as controls per Artlip *et al.*^20^ M.26 and T166 buds were also self-T-budded on their respective parent trees in order to assess whether grafting altered phenotypical or phenological aspects compared with own-rooted trees.^12,20^

### Field planting

Ten trees each of ‘Royal Gala’ on M.26 (RG/M.26) or T166 (RG/T166) rootstocks, and own-rooted M.26 and T166 trees were planted in October 2012. Ten own-budded M.26 and T166 trees were planted in October 2013. All plantings were located on the grounds of the USDA-ARS, Appalachian Fruit Research Station, Kearneysville, WV, USA. The planting design consisted of five rows, with two trees of each tree type, randomly assigned a planting location within each row. All the planted trees had been growing in a greenhouse for 6 months before the time of planting. Trees were not pruned in order to assess natural growth, however, conventional pesticide management practices were applied during the course of the study.

### Growth and phenological measurements

Growth measurements were taken monthly during the growing season (March–November). Caliper (stem diameter) data were taken at a point 20 cm above the graft union. Overall height data were taken from the tip of the main stem to the ground, and current season’s shoot length was taken from the terminal bud scar of the previous year’s growth to the tip of the current season’s growth. Dates of budbreak were recorded during spring 2013 and 2014, and expressed as Julian Day of Year. In 2013, the date of budbreak of the terminal bud on the main shoot and 20 lateral buds was recorded. In 2014, the date of budbreak of the terminal bud and 20 lateral buds of the main shoot was recorded, as well as the terminal bud and 20 laterals buds on two lateral shoots. Budbreak data were recorded on three trees each of ‘Royal Gala’ grafted on either M.26 or T166 and for own-rooted trees.

### Assessment of freezing tolerance by ion-leakage assays

Leaves from field-grown trees were harvested pre-dawn during late-June 2014, thus minimizing any impact of low temperature exposure on cold acclimation, leaf health, growth status or any interfering effect of variable leaf water status. Ion-leakage assays were performed as described in Artlip *et al.*^20^ The LT_50_ (lethal temperature at which 50% of the tissue is killed) was derived as described by Zhang and Willison.^34^ The percent leakage data from unfrozen controls were set at 0% and leakage obtained at the lowest test temperature was set at 100%, thus normalizing the data over a range of 0–100%. The data were plotted with Origin version 7.5 (OriginLab, Northampton, MA, USA) and a sigmoidal curve fitted to the plotted data. All measurements were replicated three times where a replicate represents an individual tree (*n*=9).

### RT-qPCR

Bark tissue was harvested from lateral branches of RG/ M.26, RG/ T166 and own-rooted T166 trees taken during June, July and August 2015. These months were chosen based on the growth data of the current study and that of Artlip *et al.*,^20^ and expression data reported by Wisniewski *et al.*,^12^ who found that the growth-related *MdDELLA* (*MdRGL*) genes had variable kinetics during those months. Bark was scraped directly into liquid N_2_, lyophilized and stored at −20 °C until used. Total RNA was isolated from bark tissue samples using Concert Plant RNA Reagent (Invitrogen, Carlsbad, CA, USA), treated with DNase (Turbo DNA-free Kit; Ambion, Austin, TX, USA) and then diluted based on the preliminary testing for optimal response. Reverse transcription-quantitative polymerase chain reaction (RT-qPCR) analysis was performed using appropriate quantities of total RNA (per preliminary testing) as a template with the Power SYBR Green RNA-to-Ct 1-Step Kit (Applied Biosystems, Foster City, CA, USA) and 2.0 pmol of each primer per reaction (Supplementary Table 1); no-RT control reactions were included to ensure no residual DNA contamination. A ViiA 7 Real-Time PCR System (Applied Biosystems) was set to cycle as follows: cDNA synthesis at 48.0 °C for 30 min; 95.0 °C denaturation for 10 min; 40 cycles of 95.0 °C for 15 s followed by 52.0–57.0 °C (depending on primers used) for 1 min; followed by ABI-specified hold and melt curve stages. Primers were verified for specificity by using genomic DNA template and assessing the resulting amplicon by agarose gel electrophoresis and qPCR with genomic DNA on the ViiA 7 Real-Time PCR System; all primers had a single band and single peak. Primer efficiency was also verified for all primer sets by constructing a standard curve using qPCR data. Three technical replicates were used for each of the three biological replicates (tree). The standard curve method (user bulletin no. 2; Applied Biosystems http://www3.appliedbiosystems.com/cms/groups/mcbsupport/documents/generaldocuments/ cms_040980.pdf) was used to calculate transcript abundance relative to *MdoLTL1* as a reference gene.^35^ Other endogenous reference genes were also examined, but *MdoLTL1* was determined to be the best overall reference gene using NormFinder.^36^ To weight the importance of biological variation over technical variation, technical replicates were nested within biological replicates in calculating the mean square error term. The expression of *PpCBF1* was assessed slightly differently. Rather than the final melt step in the RT-qPCR protocol previously described, reactions were separated on a 2% agarose gel, stained with ethidium bromide, and scanned on a Typhoon FLA 9500 scanner (GE Healthcare, Piscataway, NJ, USA) to determine if any and how much product was generated.

## Results

**Figure 1:**
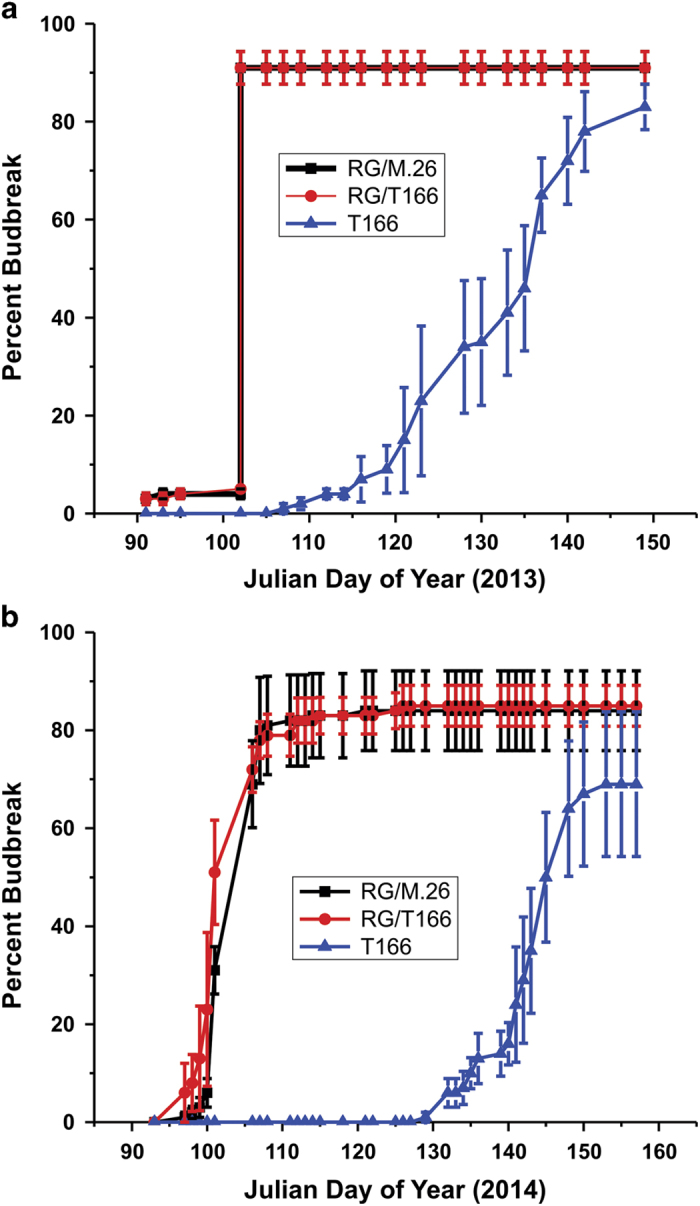
Budbreak of RG/M.26, RG/T166 and T166 trees over two seasons. (**a**) 2013 data. (**b**) 2014 data. Twenty buds basipetal to the terminal bud of the central axis were monitored for budbreak (green tip) and percent budbreak calculated. Symbols represent the mean±s.e. (*n*=360). Black squares, RG/M.26; red circles, RG/T166; blue triangles, own-rooted T166.

After establishing the planting in the fall of 2012, phenotypic measurements were commenced in the spring of 2013. Budbreak data, defined as the green tip stage in vegetative buds,^37^ is presented in Figure 1. No discernable impact of the T166 transgenic rootstock on the time of budbreak of ‘Royal Gala’ was observed. Budbreak in RG/T166 and RG/M.26 went from ~5 to 90% in a 24-h period in 2013 stimulated by the very warm temperatures (maximum temperatures⩾28 °C) that occurred for several days just before the abrupt rise in percent budbreak. The timing and rapid increase in percent budbreak was similar in 2014. This was in sharp contrast to the delayed budbreak observed in own-rooted and self-budded T166 trees.

**Figure 2:**
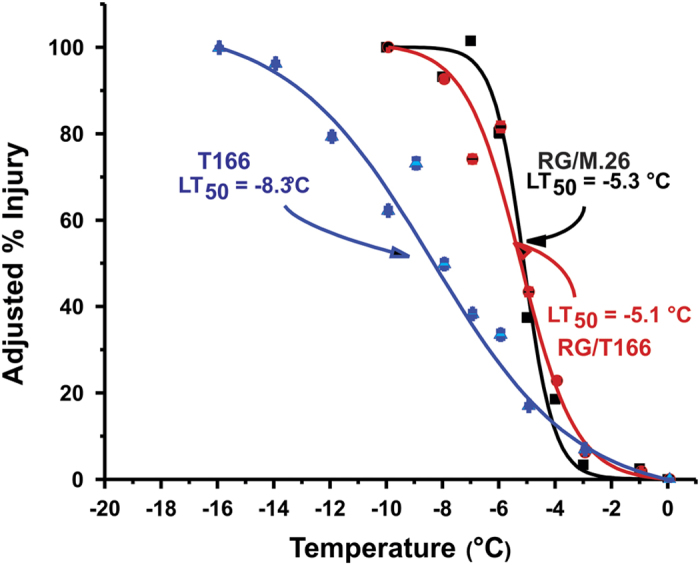
Freezing tolerance of non-acclimated (June) leaves collected from RG/M.26, RG/T166 and T166 own-rooted trees. No difference in freezing tolerance was observed between RG/M.26 and RG/T166 leaves, while T166 exhibited enhanced freezing tolerance. Black squares, RG/M.26; red circles, RG/T166; blue triangles, own-rooted T166. Symbols represent mean±s.e. (*n*=3). Each biological replicate (tree) consisted of three technical replicates.

**Figure 3:**
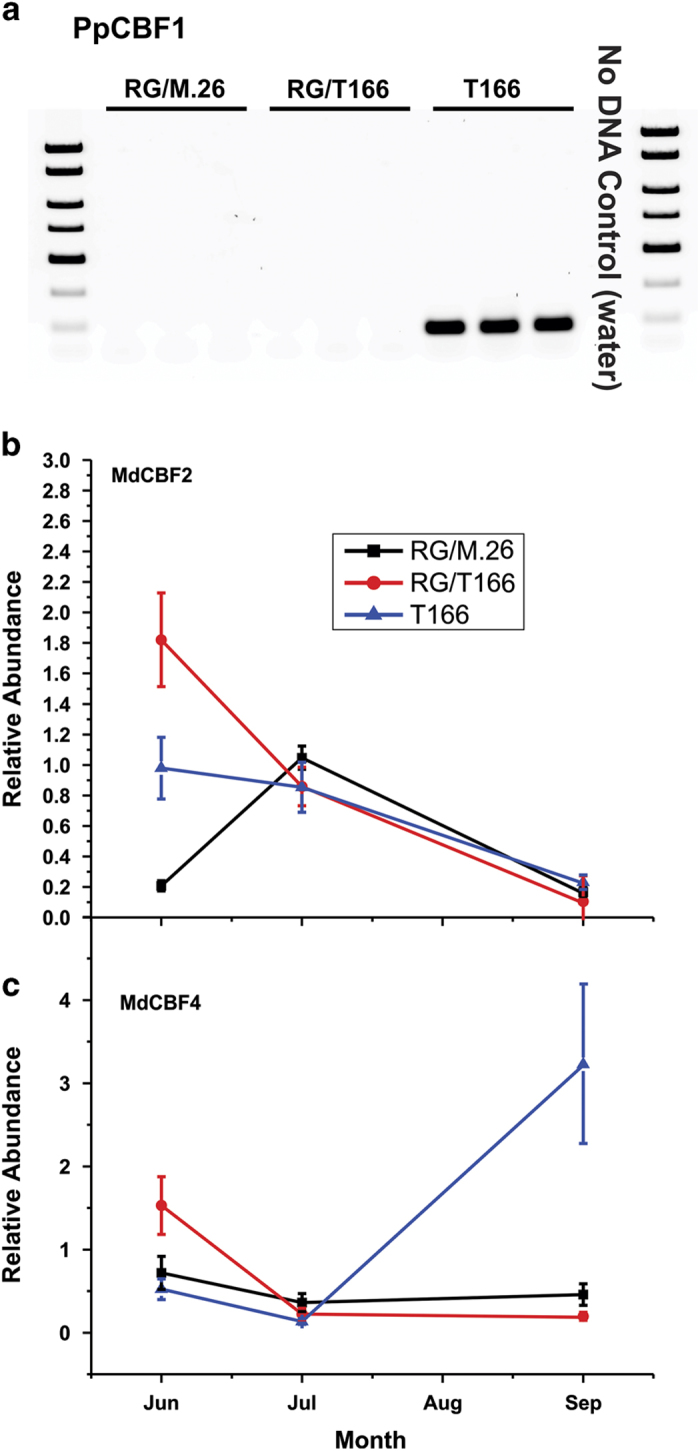
Expression of *PpCBF1* and native *MdCBF* genes in scion bark tissues of RG/M.26, RG/ T166 and T166 own-rooted trees during the summer growing season. (**a**) *PpCBF1* expression as determined by PCR. The PCR products were separated on a 2% agarose gel. (**b**) RT-qPCR of *MdCBF2*. (**c**) RT-qPCR of *MdCBF4*. Black squares, RG/M.26; red circles, RG/T166; blue triangles, own-rooted T166. Data represent the mean±s.e. (*n*=3). Each biological replicate (tree) was composed of three technical replicates.

Graft transmissibility of enhanced freezing tolerance from the transgenic T166 rootstock to the ‘Royal Gala’ scion was assessed on leaves in June 2014 by ion leakage. The LT_50_ (temperature at which 50% of the tissue is killed) in leaves of RG/M.26 versus RG/T166 were compared. Results indicated that there were no significant differences in the LT_50_ of leaves between the two types of trees (Figure 2). In contrast, leaves obtained from own-rooted T166 trees displayed a significant enhancement in freezing tolerance. No detectable level of *PpCBF1* mRNA was observed in the phloem/cambium tissues of ‘Royal Gala’ bark tissues of scions that had been grafted on to T166 rootstocks, which implies that there was no graft transmittance of transgene mRNA from the rootstock to the grafted scion (Figure 3a). The expression of two endogenous apple *CBF* genes, *MdCBF2* and *MdCBF4*, was also examined by RT-qPCR to determine whether or not *PpCBF* overexpression in the T166 rootstock influenced their expression in the RG scions (Figures 3b and c). In June, the highest level of expression of *MdCBF2* was observed in RG/T166 and T166 trees while the lowest level was observed in RG/M.26 trees. Levels of *MdCBF2* expression were similar in all three tree types in July and September. *MdCBF4* expression was generally low and similar in all three tree types but exhibited a sharp increase in T166 trees in September.

**Figure 4:**
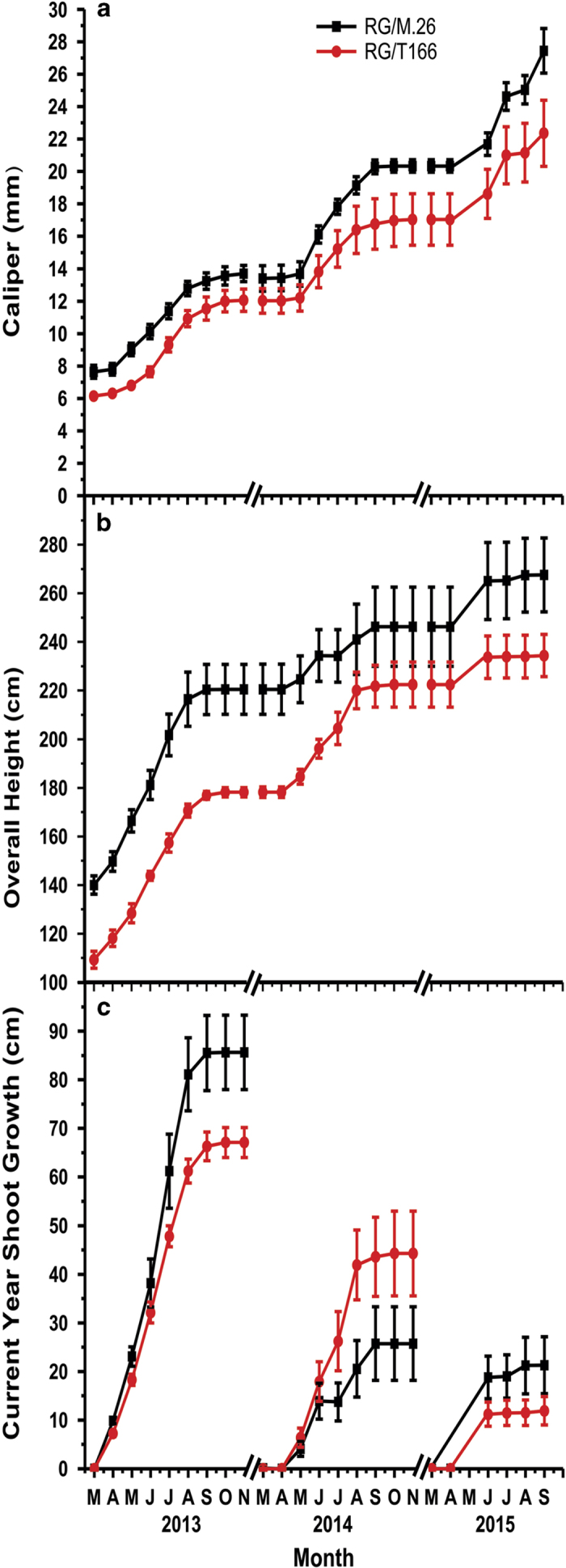
Growth of RG/M.26 and RG/T166 trees over three growing seasons. (**a**) Caliper (stem diameter) 20 cm above the graft union. (**b**) Current-year shoot growth taken from previous season’s bud scar to the shoot terminus. (**c**) Overall height. Black squares, RG/ M.26; red circles, RG/ T166. Symbols represent mean±s.e. (*n*=5).

**Figure 5:**
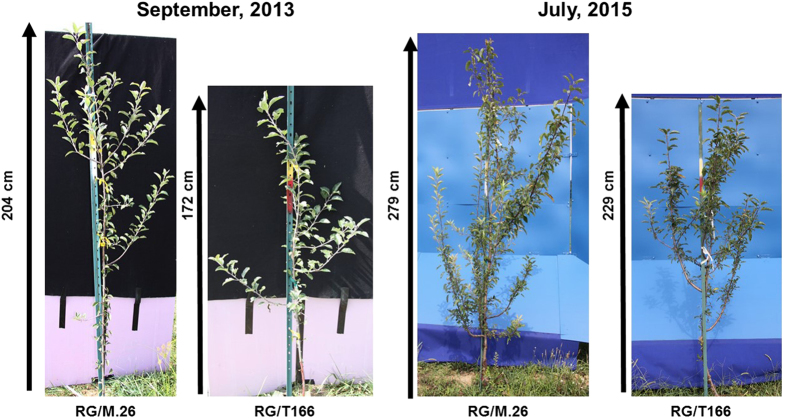
Photo of representative RG/M.26 and RG/T166 trees illustrating difference in growth in September 2013 and July 2015. Overall growth and the number of lateral branches were greater in RG/M.26 than RG/T166 trees.

Growth of RG/T166 trees and RG/M26 trees, as measured by stem diameter, current-year shoot extension and overall height, was recorded over a 3-year period (Figure 4). Increases in caliper (stem diameter) and overall height in RG/T166 trees were consistently less than in RG/M.26 trees in all three growing seasons (Figures 4a and b). Differences between the trees at the onset of the measurements reflect the status of trees after growing in the greenhouse for ~6 months. Current-year shoot growth in RG/ T166 trees was significantly less than in RG/M.26 in both 2013 and 2015 but not 2014 (Figure 4c). Field notes indicate, however, that several of the tagged branches used to measure current-year shoot growth in RG/M.26 trees exhibited death of a terminal bud. As the sub-terminal buds did not grow appreciably after the death of the terminal bud, this impacted the recorded overall current-year shoot growth in 2014. No obvious reason for the death of the terminal buds was evident. Representative photos illustrate the growth disparity between the tree types (Figure 5). Differences in the number of lateral branches present in the two tree types can also be observed in Figure 5. RG/ M.26 trees generally had a higher number of lateral branches than RG/ T166 trees.

**Figure 6:**
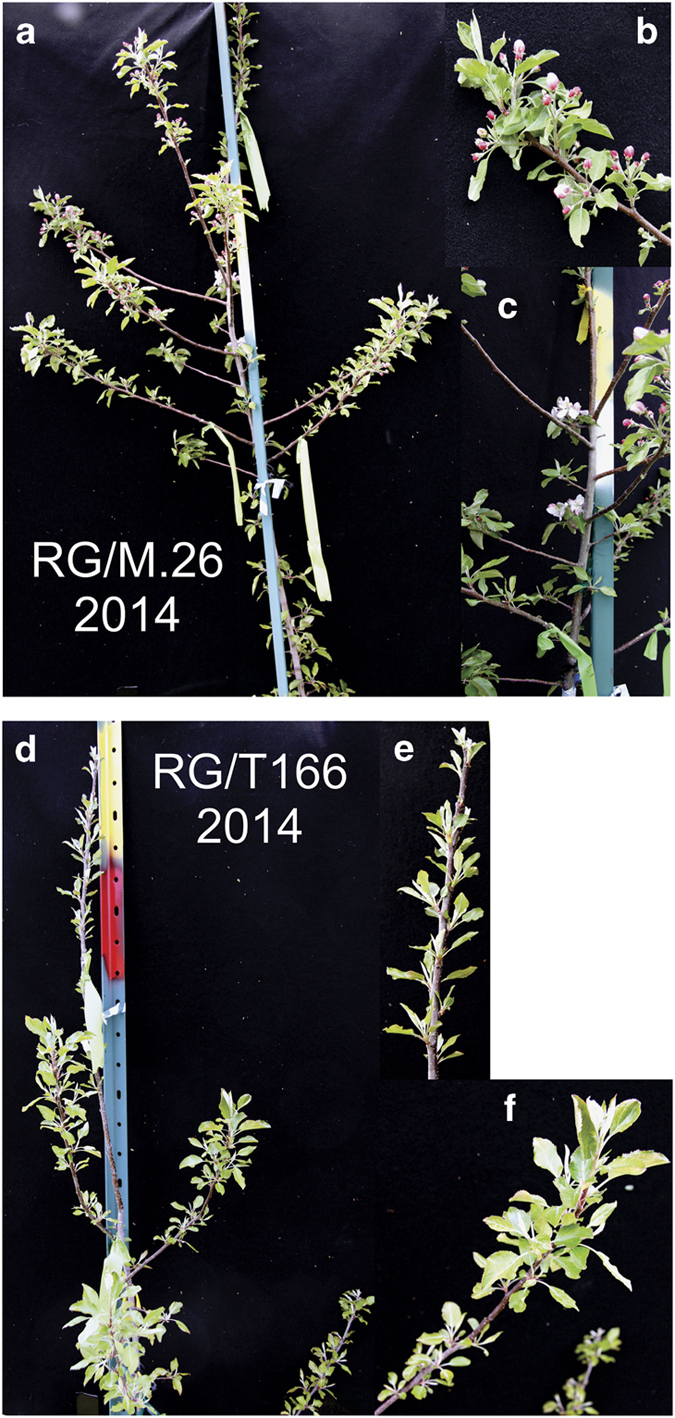
Photo of representative of RG/M.26 and RG/T166 trees in the spring of 2014 illustrating differences in the level of flowering in the two tree types. A greater number of RG/M.26 trees had floral bud clusters than RG/T166 trees. (**a**) Representative RG/M.26 tree. (**b** and **c**) Close up photos of a RG/M.26 tree showing numerous floral bud clusters. (**d**) Representative RG/T166 tree. (**e** and **f**) Close up photos of a RG/T166 tree showing no floral bud clusters.

**Figure 7:**
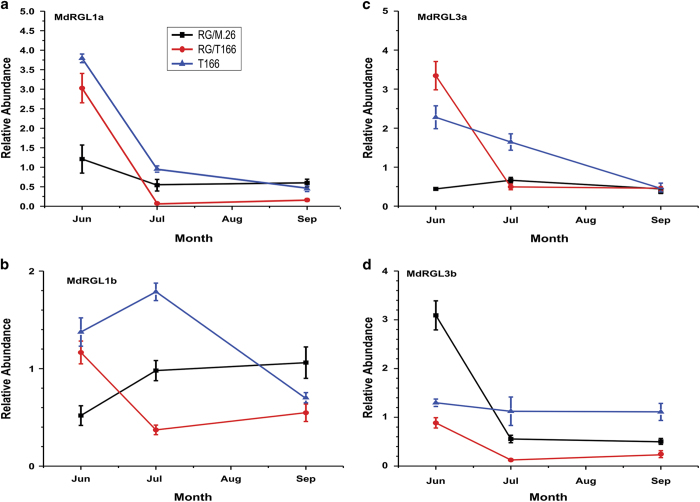
RT-qPCR analysis of *MdRGL* gene expression in bark tissues of scions from RG/M.26, RG/T166 and T166 own-rooted trees during the summer growing season. (**a**) *MdRGL1a*. (**b**) *MdRGL1b*. (**c**) *MdRGL3a*. (**d**) *MdRGL3b*. Black squares, ‘RG’/M.26; red circles, ‘RG’/T166; blue triangles, own-rooted T166. Symbols represent mean±s.e. (*n*=3). Each biological replicate (tree) was composed of three technical replicates.

The RG/M.26 and RG/T166 trees began to flower during the second growing season in the field, 2014 (Figure 6). The number of trees with bloom clusters and the average number of bloom clusters per tree differed markedly between the RG/M.26 and RG/ T166 trees in 2014 and subsequently in 2015. RG/M.26 trees consistently exhibited greater levels of flowering than RG/T166 trees (Table 1). Earlier research reported that overexpression of *PpCBF1* resulted in both reduced growth and the overexpression of two *RGL* (*DELLA*) genes, *MdRGL3a* and *MdRGL3b*.^12^ In general, *DELLA* genes have been associated with a suppression in GA responses. In the current study, the expression of four endogenous *RGL* (*DELLA*) genes was examined during June, July and September 2015, the time period of very active growth (Figure 4). Although *RGL* expression levels varied over this time period, levels of *MdRGL 1a*, *1b* and *3a* expression in RG/T166 and own-rooted T166 were similar in June and contrasted with the level of *RGL* expression in RG/M.26 trees (Figure 7). Subsequently, the pattern of *RGL* expression in July and September was similar in all three tree types. In general, *RGL* expression decreased in all three tree types over the measured time course. The only exception to the stated patterns was for *MDRGL3b* when levels of expression were significantly higher in RG/M.26 trees than in the other two tree types (T166 and RG/T166).

## Discussion

Previous studies of overexpression of a peach CBF gene (*PpCB1)* in M.26 apple rootstocks revealed a significant impact on several developmental and phenological parameters.^10,12,20^ Transgenic trees (line T166) exhibited increased levels of non-acclimated and acclimated freezing tolerance, response to short photoperiod leading to the early onset of dormancy and early leaf senescence, and delayed budbreak, relative to non-transgenic M.26 trees. A reduction in overall height, stem caliper and current-year shoot growth was also observed in T166 trees. This was true in both greenhouse and several years of field studies.^10,12,20^ As rootstocks are typically used to control growth and impart other horticultural traits in scion varieties,^26^ the purpose of the present study was to determine if the physiological traits observed in T166 trees could be imparted to or at least influence scions that were grafted on to T166 rootstocks.

In previous studies and the current study, own-rooted T166 trees overexpressing the *PpCBF1* gene exhibited early dormancy, early leaf senescence and delayed spring budbreak compared with non-transgenic M.26 trees.^10,12,20^ These physiological and phenological attributes, however, were not observed in the scion (Royal Gala) that was grafted on the T166 rootstock. No major differences were observed between RG/T166 and RG/M.26 trees in any of these parameters. In addition, no differences were observed in non-acclimated leaf-freezing tolerance between RG/T166 and RG/M.26 trees. These results clearly indicate that it would not be possible to use this transgenic rootstock to convey the dormancy and freezing tolerance attributes exhibited by the transgenic rootstock to different scion genotypes grafted on to this rootstock. Transmission of mRNA from herbaceous transgenic donor plants to recipient plants through a graft union via the phloem have been documented.^27^ No evidence of *PpCBF1* mRNA, however, was observed in the ‘Royal Gala’ scions that had been grafted on T166 transgenic rootstocks (Figure 3a). Although elevated levels of endogeneous *MdCBF2* were present in the RG/T166 trees in June, relative to both RG/M.26 trees and T166 trees, when freezing tolerance was assayed, no impact on freezing tolerance was observed in the RG/T166 trees (Figure 3b). In contrast, *MdCBF4* levels were greatly elevated in September only in the T166 trees (Figure 3c). These data suggest that *MdCBF2* is perhaps more related to water deficit and responsible for the different levels of expression observed between the tree types. In contrast, *MdCBF4* may be more related to freezing tolerance, thus becoming greatly elevated in the T166 September samples due to fact that the T166 trees had entered dormancy and responded to a shortening photoperiod while the other tree types (RG/M.26 and RG/T166) had not. In this regard, leaves serve as the point of perception of photoperiod.^38^ In the present study, transgenic rootstock leaves were not present on the RG/T166 trees. This could account for the lack of impact of the rootstock on the various dormancy attributes in the scion in RG/T166 trees. Without transgenic leaves present, the rootstock could not respond to the shortening photoperiod and thus impact dormancy the way it does in own-rooted T166 trees. Trees possessing branches and leaves derived from both scion bud grafts and the T166 transgenic rootstock are presently being developed to test this hypothesis.

Despite the lack of impact of the T166 transgenic rootstock on scion dormancy and freezing tolerance, a consistent and significant effect was observed on scion growth and precocity, as evidenced by reduced growth and flowering in RG/T166 trees (Figures 4, 5, 6 and Table 1). Stem caliper, current-year shoot growth (except for 2014), and overall tree height was also reduced in RG/T166 trees relative to RG/M.26 trees (Figure 4). In addition, beginning in 2014, the number of trees with flowers was greater in RG/M.26 trees than it was in RG/T166 trees, as was the number of branches with flowers, and the total number of flower clusters.

Constitutive overexpression of *CBF* genes has been reported to result in diminished growth in several plant systems. Herbaceous plants such as *Arabidopsis*,^17–19,39^ potato^14^ and tomato^13^ have exhibited this phenotype when native or exogenous *CBF* genes were overexpressed. Woody plants, such as *Eucalyptus*,^8^ birch,^5^ poplar,^6^ grape^15^ and apple^10,12,20^ have also exhibited reduced growth with *CBF* gene overexpression. The reports in apple demonstrated that the reduced growth occurs not only in greenhouse and environmental chamber studies but over the course of several years when trees are planted in the field.^10,12,20^ Growth reduction on the order of 20% (stem diameter) and 13% (tree height) was seen in the current study in RG/T166 trees after three growing seasons, compared with RG/M.26 trees (Figure 4). Growth reduction was even more pronounced in own-rooted or own-grafted T166 trees, being reduced by 25% in stem diameter and 33% in overall height) after three growing seasons, compared with own-rooted M.26 trees (Supplementary Figure S1).

The disparity between RG/ T166 and RG/ M.26 trees extended beyond overall height and stem diameter as RG/ T166 trees also produced fewer lateral branches (Figure 5). Artlip *et al.*^20^ and Wisniewski *et al.*^12^ noted a similar trend when comparing field-grown, own-rooted T166 with M.26 trees.

Rootstocks are known to affect various scion traits, such as growth and tree architecture.^40^ The question arises, however, as to how the overexpression of a *CBF* gene can further the impact of a specific rootstock on the horticultural traits of a grafted scion cultivar. In regards to growth, Wisniewski *et al.*^12^ reported on the impact of *CBF* on the expression of *RGL* (*DELLA*) genes, which are generally considered to inhibit GA-mediated growth processes.^41^ They were able to demonstrate higher levels of expression of at least two native apple *RGL* genes and suggested that the high levels of these genes resulted in the inhibition of GA-mediated growth at the time of year when the rate of growth was greatest. Higher expression levels of three native *RGL* genes (*MdRGL1a*, *1b* and *3a*) were observed in the present study in RG/T166 and own-rooted T166 trees, compared with RG/M.26 trees. These high levels were observed in the month of June when growth rates were high. Overexpression of CBFs have been shown to decrease GA levels and increases DELLA levels in other plant systems.^23,42,43^ Moreover, Xu *et al.*^44^ demonstrated that *RGL* mRNA is phloem-mobile and that both acropetal and basipetal translocation of mRNAs of two *GAI* (=*RGL*=*DELLA*) genes can occur across the graft union of ‘Fuji’/*Malus xiaojinensis* trees. The combination of CBF effects on bio-active GAs, DELLAs and the reports on translocation across graft unions provide a framework to explain the observations reported in the current study.

In addition to the influencing scion growth and architecture, rootstocks are also known to effect time to flowering or precocity.^40^ In the present study, time to flowering was delayed in RG/T166 trees compared with RG/M.26 trees (Table 1). Although flowering commenced in RG/M.26 trees in the second season of growth (2014), flowering was very sparse in RG/T166 trees. This difference persisted in subsequent seasons with RG/M.26 trees exhibiting a greater number of trees with flowers, more branches with flowers on individual trees, and more flower clusters per flowering shoot (Figure 6; Table 1). The influence of the T166 rootstock on precocity and number of floral bud clusters is perhaps unexpected, given that dwarfing rootstocks tend to result in earlier bearing than semi-dwarfing or standard rootstocks,^26^ and T166 is clearly more dwarfing than M.26. However, Gilmour *et al.*^40,41^ also reported that overexpression of native CBF genes in *Arabidopsis* inhibited flowering. Therefore, the relationship between CBF overexpression and inhibition of flowering is not unprecedented. Nonetheless, the observations in the present study are in contrast to the general impact of dwarfing on flowering. Watson *et al.*^40^ in a study on the effects of quince (dwarfing) and *Pyrus calleryana* (vigorous) on pear scions, reported that the least vigorous rootstocks produced the highest number of floral buds. In this regard, Fazio *et al.*^26^ reported that the Quantitative Trait Locus or Loci (QTL) for early bearing co-located with the QTL for scion vigor suppression (dwarfing). Therefore, the relationship between low vigor and precocity would be expected due to linkage disequilibrium.

**Figure 8:**
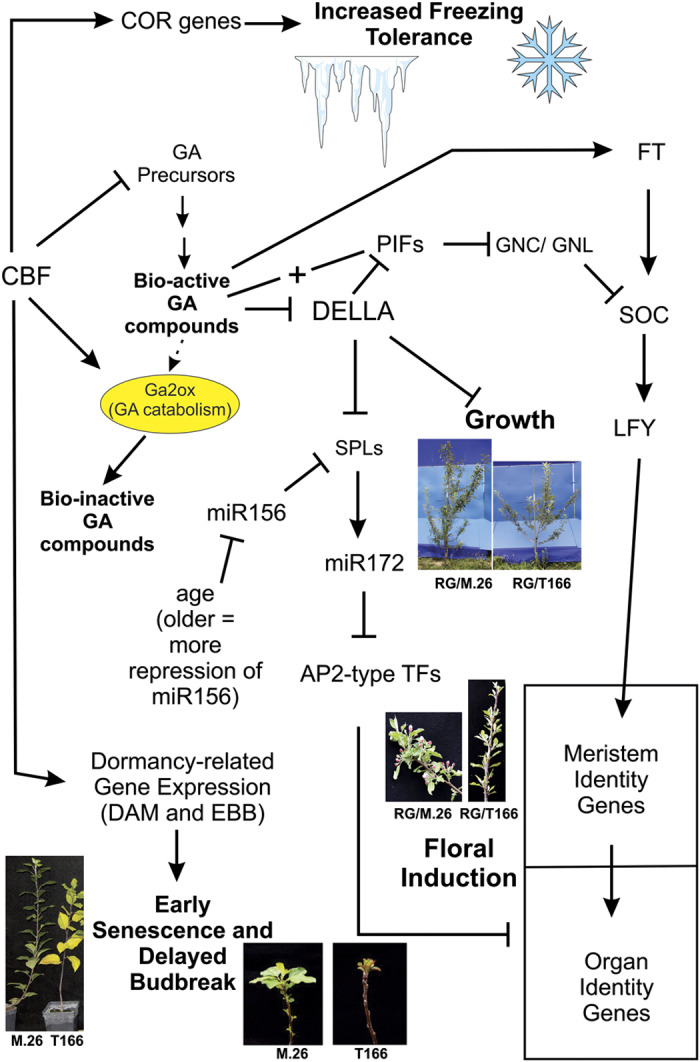
Schematic diagram of the potential influence of *CBF* gene expression on the regulation of freezing tolerance, dormancy, growth and juvenility (time to flowering). In the model, *CBF* gene expression regulates *RGL* (*DELLA*) gene expression which then impacts expression of growth and flowering-related pathways. *CBF* gene overexpression directly affects freezing tolerance and dormancy. Solid lines with arrowheads indicate positive regulation; solid lines ending as a ‘T’ indicate negative regulation; dotted line with arrowhead indicates GA inactivation pathway; ‘**+**’ indicates interaction of GA with PIF. AP-2, APETALA-2; COR, cold regulated; EBB, early budbreak; GNC/GNL, GNC: GATA, nitrate-inducible, carbon-metabolism involved; GNL, GNC-like; GAMYB, GA-regulated MYB transcription factor; miR156 and miR172, micro RNAs; TFs, transcription factors. Figure based on models presented in various reports.^12,22,23,41,45–48,51–55^

In the case of overexpression of *PpCBF1* in the rootstock resulting in reduced growth and delayed flowering, the cause must be due to some commonality between the two processes rather than from some random effect on genes located in the region of the QTLs for either dwarfing or early bearing. This is supported by the fact that several independent *PpCBF1* overexpressing lines, representing separate random insertion events, all resulted to a similar degree in the same apple phenotype.^10^ As previously noted, Wisniewski *et al.*^12^ demonstrated that overexpression of *PpCBF1* in M.26 apple resulted in elevated levels of *RGL* (*DELLA*) genes during the time period when growth rate is very high and that these two factors (overexpression of *PpCB1* and *RGL* genes) were associated with reduced growth. It is also known that CBF overexpression can reduce the level of GA precursors^43^ and possibly increase the catabolism of bio-active GAs through GA2ox.^23^ In addition to their known growth-repressive activities, DELLA (RGL) proteins have also been reported to affect several portions of floral induction pathways. A schematic diagram of how CBF may influence flowering and other developmental parameters is presented in Figure 8. This schematic expands on the model presented by Wisniewski *et al.*^12^ illustrating how CBF overexpression could modulate freezing tolerance, dormancy, growth and indirectly floral induction. DELLA levels antagonize SPL (Squamosa promoter binding like) proteins, which positively regulate one of the pathways of floral induction.^22^ SPLs are a component of the miR156 (micro RNA 156) mediated-age pathway in *Arabidopsis*, with SPL levels gradually increasing during the transition from the juvenile to adult states.^45^ Specific phytochrome interacting factors (PIFs) integrate light and GA signals to regulate another floral induction pathway. Downstream of the PIF/GA interaction are GNC/GNL proteins (GNC: GATA, nitrate-inducible, carbon-metabolism involved; GNL: GNC-like), which act as repressors of the flowering time agent, SOC (suppressor of overexpression of constans 1), in *Arabidopsis.*^46^ DELLAs act via repression of PIF, thus maintaining repression of floral (and other developmental pathways) by GNC/GNL.^45,46^ SOC, is a known component of a floral induction pathway including CO (constans), FT (flowering time) and the floral meristem identity protein, LFY (leafy)^47^. In addition, decreased levels of bio-active GAs may contribute toward reduced levels of *FT*.^47,48^ Apple homologs of *SPL* and *GNC*/*GNL* genes were identified by BLAST searches of the apple genome in the Genome Database for Rosaceae (GDR, http://www.rosaceae.org/) (data not shown).^49,50^

A potential argument against the model presented in Figure 8 is that in some studies GA has been reported to inhibit floral induction in apple, especially when exogenous GA is applied.^51,52^ However, in a review of the available literature, Bangerth^52^ indicated that ambiguities exist when regarding the effects of endogenous GA on floral induction in fruit trees.

In conclusion, the current study examined the ability of transgenic T166 rootstocks to impact various physiological and developmental properties of ‘Royal Gala’ which was used as a scion and grafted on the T166 rootstock. Dormancy, freezing tolerance, growth and flowering data were recorded for RG/T166 and RG/M.26 trees and compared with each other and with data recorded for own-rooted and self-grafted M.26 and T166 trees. Dormancy (as measured by budset, leaf senescence and spring budbreak) and freezing tolerance were similar in RG/T166 and RG/M.26 trees. No evidence of graft transmission of the *PpCBF1* transgene was observed. In contrast, both growth and precocity (time to flowering) were inhibited and delayed, respectively, in RG/T166 trees compared with RG/M.26 trees. Elevated levels of *DELLA* (*RGL*) mRNA in scions grafted to transgenic rootstocks may partially account for the observed differences in growth and flowering.

## Figures and Tables

**Table 1:** Percent of trees in flower and the average number of bloom clusters per tree in 2014 and 2015 in RG/M.26 and RG/M.26 trees

*Scion/rootstock*	*Year*	*Percent of trees with bloom clusters (*n*=10)*	*Average number of bloom clusters per tree (*n*=10)*
RG/M.26	2014	100	10
RG/T166	2014	20	0.5
RG/M.26	2015	100	103.5
RG/T166	2015	90	31.6

Percent and average number of blooms per tree are based on a total of 10 trees for each tree type.
