# Supplementary material for: An apple rootstock overexpressing a peach CBF gene alters growth and flowering in the scion but does not impact cold hardiness or dormancy
Source: Hortic Res. 2016 Mar 9;3:16006–. doi: 10.1038/hortres.2016.6 (PMC4783695; doi:10.1038/hortres.2016.6)
Supplement: Supplementary Information [file hortres20166-s1.doc]

Supplementary Table 1. Primers used for RT-qPCR

| **Gene** | **For (5' - 3')** | **Rev (5' - 3')** | **GDR accession number** |
| --- | --- | --- | --- |
| **PpCBF1** | **gcacattgtggatatgggaaaaag** | **gggttggggtggagaaagaag** | **ppa014628m (*Prunus persica* v1.0)** |
| **MdCBF2** | **tctccgcctcactcttca** | **gaaagcgtccgaaagtt** | **MDP0000198054** |
| **MdCBF4** | **actgggatgatatgggaa** | **gtgtcctttagggatgat** | **MDP0000154764** |
| **MdRGL 1A** | **agaacgacgggtgtatga** | **cacccaaaaaaaatggaaccga** | **MDP0000237978** |
| **MdRGL 1B** | **ctcattgccacctcggctt** | **aaccccaccaccatcacca** | **MDP0000640034** |
| **MdRGL 3A** | **cacaatcaaccaccaaac** | **gaagtaacacaaagagcaag** | **MDP0000662303** |
| **MdRGL 3B** | **gaagccacggcaaaccaa** | **cattaaggtagaacacagac** | **MDP0000134341** |
| **MdLTL1** | **ccatatccaggcttgcctaa** | **acgagggtaacctcacatgc** | **MDP0000173025** |
| **MdEF1-α** | gacattgccctgtggaagtt | ggtctgaccatccttggaaa | MDP0000487255 |
| **MdCKB4** | gttgaatcagcagcggagat | aaactcttgggcatcttcca | MDP0000095375 |
| **MdFYPP3** | tcgagcacaccaacttgttc | gatagaagccacattcccaca | MDP0000060858 |

Table S1 Legend:

| Gene names include Genome Database for Rosaceae (<https://www.rosaceae.org/>) predicted transcript accession numbers. *LTL1, StaR-related lipid transfer protein*; *EF1-α*, *transla­tion elongation factor; FYPP3, Phytochrome protein phosphatase 3; MdCKB4,* *Casein kinase II subunit beta-4*. Non-bold font indicates endogenous reference genes that were found unsuitable due to insufficient stability across time. All GDR accession numbers from the *Malus* x *domestica* v1.0 genome except where noted.  **Supplementary Figure 1**. Growth of own-rooted and own-grafted trees over three growing seasons. ‘M.26 trees typically displayed greater growth than T166 trees, regardless of graft status. a. Caliper (Stem Diameter) 20 cm above the graft union. b. Current Year Stem Growth taken from previous season’s bud scar to the terminus. c. Overall Height. Black squares, M.26 own-rooted (planted Oct., 2012); red circles, T166 own-rooted (planted Oct., 2012); Blue triangles, M.26 own-grafted (Planted Oct., 2013); Green inverted triangles, T166 own-grafted (planted Oct., 2013). Symbols represent means ± SE (n=5 biological replicates). |
| --- |
